# Cortisol Is an Associated-Risk Factor of Brain Dysfunction in Patients with Severe Sepsis and Septic Shock

**DOI:** 10.1155/2014/712742

**Published:** 2014-04-28

**Authors:** Duc Nam Nguyen, Luc Huyghens, Haibo Zhang, Johan Schiettecatte, Johan Smitz, Jean-Louis Vincent

**Affiliations:** ^1^Department of Critical Care Medicine, Universitair Ziekenhuis Brussel, Vrije Universiteit Brussel, Laarbeeklaan 101, 1090 Brussels, Belgium; ^2^Department of Anesthesia and Critical Care Medicine and Keenan Research Centre for Biomedical Science, St. Michael's Hospital, Bond Street 30, Toronto, ON, Canada M5B 1W8; ^3^Department of Immunochemistry, Universitair Ziekenhuis Brussel, Vrije Universiteit Brussel, Laarbeeklaan 101, 1090 Brussels, Belgium; ^4^Department of Intensive Care, Erasme University Hospital, Université Libre de Bruxelles, Route de Lennik 808, 1070 Brussels, Belgium

## Abstract

*Objectives*. To investigate cortisol levels in brain dysfunction in patients with severe sepsis and septic shock. *Methods*. In 128 septic and sedated patients, we studied brain dysfunction including delirium and coma by the evaluation of Richmond Agitation Sedation Scale (RASS), the Confusion Method Assessment in the ICU (CAM-ICU) after sedation withdrawal and the measurement of serum S100B biomarker of brain injury. Serum cortisol and S100B were measured within 12 hours after ICU admission and daily over the next four days. *Results*. Brain dysfunction was observed in 50% (64/128) before but in 84% (107/128) of patients after sedation withdrawal, and was more common in the patients older than 57 years (*P* = 0.009). Both cortisol (*P* = 0.007) and S100B levels (*P* = 0.028) were higher in patients with than patients without brain dysfunction. Cortisol levels were associated with ICU mortality (hazard ratio = 1.17, *P* = 0.024). Multivariate logistic regression showed that cortisol (odds ratio (OR): 2.34, 95% CI (2.01, 3.22), *P* = 0.02) and the combination effect of cortisol with age (OR: 1.004, 95% CI (1.002, 1.93), *P* = 0.038) but not S100B were associated with brain dysfunction. *Conclusions*. Cortisol was an associated-risk factor of brain dysfunction in patients with severe sepsis and septic shock.

## 1. Introduction

Cortisol release from the hypothalamic-pituitary-adrenal axis (HPA) is vital for the host survival in stress [1]. In sepsis, high cortisol release could result not only from stress but also from HPA dysfunction and following a metabolism reduction of this hormone at the organs [2–4]. However, an excessive release or chronic exposure to high cortisol levels could be harmful for the host brain, especially at the hippocampus and the frontal cortex where corticoid receptors are highly concentrated. It has been shown in rodents' experimental studies that cortisol reduced the neurons viability to toxic insults (glutamate, hypoxia, ischemia, or hypoglycemia) by ATP energy deprivation in these parts of the brain [5, 6].

Cortisol has been suggested as biomarker for the diagnosis of delirium [7]. High cortisol release associated with delirium was previously reported in stroke [8], in postcardiac surgery [9], in the elderly patients with hip fracture [10], in psychological depression, and in Cushing syndrome [11]. In clinical severe sepsis, high cortisol levels have been reported in patients who developed fatal brain dysfunction [12] but it remained unclear if this could be a sole indicator of severe inflammation and disease severity or was due to brain dysfunction. Also, these results were only reported in a small number of patients with severe sepsis and remain to be confirmed in a larger study sample.

In this observational prospective study, we investigated the effect of cortisol on the development of brain dysfunction in patients with severe sepsis and septic shock. Brain dysfunction including coma and delirium was evaluated by the Glasgow Coma Score (GCS) or the Richmond Agitation Sedation Scale (RASS) combined with the Confusion Assessment Method in the ICU (CAM-ICU) and with the measurement of serum biomarker of brain injury S100B protein [13].

We also compared the predictive value of cortisol with S100B on the development of brain dysfunction as the measurements of serum biomarkers (S100B protein, neuron-specific enolase, and glial fibrillary acidic protein) have been proposed to help diagnose brain dysfunction regardless of sedation [14].

## 2. Patients and Methods

We studied 140 patients with severe sepsis and septic shock who were consecutively included in the study from October 2009 to January 2011. The Ethical Committee of our hospital approved the study and the informed consent was obtained from the patient's relatives.

Severe sepsis and septic shock was considered before ICU admission and the inclusion criteria were defined following the international consensus guidelines of sepsis [15]. Exclusion criteria included patients younger than 18 years old, pregnancy, acute cerebral disorder (trauma, stroke, hemorrhage, post-neurosurgery, cardiorespiratory arrest, and meningitis), concomitant treatment with corticoid or etomidate in the previous 24 hours, drugs or alcohol withdrawal, severe psychiatric disorder, dementia with disable neuromuscular disorders, severe chronic liver or renal failure, and nonsurvivors in the first 24 hours from sepsis.

Early goal-directed therapy for sepsis resuscitation and lung protective ventilation strategy were conducted following the international consensus sepsis guidelines. Resuscitation was targeted to obtain a mean arterial pressure ≥ 65 mm Hg and a urine output > 0.5 mL/kg/min with both colloid and crystalloid infusion, combined with norepinephrine (up to 1 *μ*g/kg/min) and dobutamine (up to 10 *μ*g/kg/min). Mechanical ventilation targeted a tidal volume of 6-7 mL/kg ideal body and a plateau pressure < 30 cm H_2_O.

Sedation in the ICU was achieved by midazolam (up to 0.05 mg/kg/h) or propofol (up to 10 mg/kg/h) and analgesia was achieved by fentanyl (up to 0.05 mg/kg/min) or remifentanil (up to 0.75 *μ*g/kg/h). Sedation and analgesia doses were daily adapted to obtain a Richmond Agitation Sedation Scale (RASS) score between 0 and −3. Neuromuscular blocking agent administration was interrupted within the first 48 hours after ICU admission.

Organ failure and the critical illness severity were evaluated by the daily Sequential Organ Failure Assessment (SOFA) and the APACHE III scores, respectively.

### 2.1. Brain Dysfunction Assessment

Before sedation, brain dysfunction was assessed by either GCS or CAM-ICU and it was considered when the GCS ≤ 13 or CAM-ICU was positive. After 24 hours of complete sedation withdrawal and up to ICU discharge, CAM-ICU was assessed together with the RASS score twice per day by the nurse or the physician in charge of the patient.

Delirium was confirmed by the physician in charge of the patient who was not aware about the cortisol and S100B results when RASS score > −3 and CAM-ICU was positive for at least two consecutive days. Hyperactive delirium was defined as the RASS score of 1 to 5 with agitation, irritation, confusion, and emotional labiality. Hypoactive delirium was defined as the RASS of −2 to −1 with apathy, decreased responsiveness or movement, and low consciousness. Coma was defined as the RASS score ≤ −4 throughout the ICU stay. Acquired critical illness neuromyopathy (CINM) was confirmed by electromyography.

### 2.2. Cortisol and S100B Protein Measurement

After ICU admission and sepsis confirmation, serum cortisol and S100B were simultaneously collected not at fixed time between 6 and 12 hours after hemodynamics stabilization and then once daily in the morning for four consecutive days.

Total serum cortisol concentration was measured in all patients by radioimmunoassay (Diasorin, Stillwater, USA). Normal values in the morning range (7–10 a.m.) were 171– 535 nmol/L without gender difference. The within run coefficient was <5% and the between run coefficients of variation were <10% (range: 27 to 1650 nmol/L).

Serum S100B protein was obtained in 75 patients by radioimmunoassay (immunoradiometric assay, Roche Diagnostics GmbH, Germany) and the normal value provided by the commercial kit was ≤ 0.105 *μ*g/L.

### 2.3. Statistical Analysis

The parametric statistical methods were used with SPSS version 20.0 (SPSS, Chicago, IL) and SAS version 9.3 (SAS Institute, North Carolina, USA) for analysis.

Chi-square test or Fischer's exact test was used when appropriate for comparisons of categorical variables. Logarithm transformation was used to assume a normal distribution for continuous variables. Student's* t*-test was used for comparisons of cortisol between groups. Linear mixed models for repeated measures (including a random intercept effect and adjusting with age and gender) were used to compare the global difference of biomarkers over 4 days of measurement between groups. Nonparametric Mann-Whitney* U* test was used for comparisons of skewed values of S100B between groups.

The Pearson or Spearman correlation test was used to evaluate the correlation between continuous variable when appropriate.

Kaplan-Meier analysis with log-rank test was used to analyze the ICU survival time. The Cox proportional hazards regression model adjusted with stepwise selection of covariates (SOFA score at the first 4 days, age, cortisol levels at admission, S100B levels at day 2, and 2 binary variables: occurrence of brain dysfunction or nosocomial infection) was used to determine the risk factors associated with ICU mortality.

Multivariate binary logistic regression model adjusted with stepwise selection of covariates (cortisol levels at admission, age, S100B levels at day 2, the SOFA score at the first 4 days, gender, the length of sedation, and one interaction term (age x cortisol levels at admission)) was used to determine the associated-risk factors for the development of brain dysfunction. Variables with univariate chi-square value <0.2 were added and retained at *P* ≤ 0.05. The receiver operating characteristic (ROC) curve and the area under the curve (AUC) were calculated for the logistic regression model or to determine for the cut-off value of cortisol and age associated with delirium. Smaller Akaike criteria value was used for the selection between different logistic regression models.

Statistical significance was considered at two-sided *P* value <0.05.

## 3. Results

Of the 140 patients with severe sepsis and septic shock, 12 (9%) who died under sedation without proper neurological evaluation were excluded from analysis.

Brain dysfunction developed in 50% (64/128) of patients before sedation but up to 84% (107/128) after sedation withdrawal. Delirium developed in 85/107 patients (80%): 41 hypoactive (48%) and 44 hyperactive (52%). Twenty-two patients (20%) remained comatose: brain CT scan showed a hemorrhage in five, a stroke in three and septic emboli in one.

In delirium patients, brain CT scan was negative in 15/44 hyperactive delirium patients but showed a stroke in two and a hemorrhage in one out of 21/41 hypoactive delirium patients.

### 3.1. The Patients' Characteristics and Comorbidities at ICU Admission (Table 1)

Only difference in age was found with regard to brain dysfunction after sedation withdrawal. However, age was not different in regard to brain dysfunction occurring before starting of sedation. Patients who developed brain dysfunction were older than the other patients (67 ± 13 versus 58 ± 18 years, *P* = 0.009). Also comatose patients were older than delirium patients (72 ± 9 versus 65 ± 13 years, *P* = 0.007). As expected, ICU nonsurvivors were older than survivors (68 ± 13 versus 62 ± 14 years, *P* = 0.035).

### 3.2. Brain Dysfunction and ICU Evolution (Table 2)

When compared with non-brain dysfunction, patients who developed brain dysfunction had higher degree of SOFA score at the first four days, higher incidence of nosocomial infection and septic shock recurrence, and higher prevalence of acquired critical illness neuromyopathy (CINM) but a trend of prolonged ICU length of stay (15 ± 10 versus 23 ± 18 days, *P* = 0.06).

Brain dysfunction was associated with higher ICU mortality: death occurred in 41% (18/44) hyperactive delirium, 68% (28/41) hypoactive delirium, and all (100%) of 22 comatose patients (*P* < 0.001) but 14% (3/21) in patients without brain dysfunction. The median (interquartile) ICU survival time was 36 (14, 48) days for delirium and 8 (7, 15) day for comatose patients (all log-rank tests, *P* < 0.01).

The Cox proportional hazards regression model identified three risk factors independently associated with ICU mortality: SOFA score at the first 4 days (hazard ratio (HR) = 1.22, *P* = 0.05), cortisol levels at admission (HR = 1.17, *P* = 0.024), and occurrence of brain dysfunction (HR = 4.89, *P* = 0.001).

### 3.3. Cortisol and S100B Protein Levels in Brain Dysfunction

**Figure 1:**
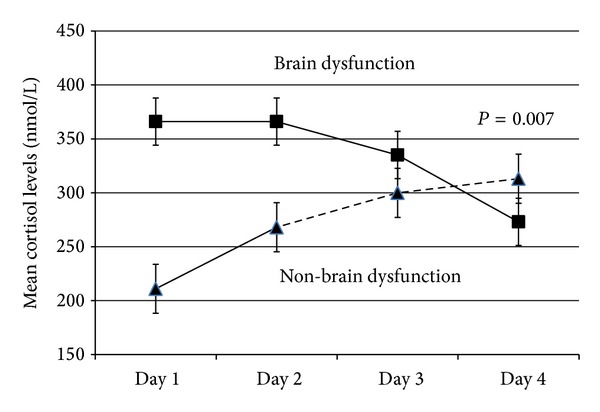
Patients with brain dysfunction released higher cortisol levels than non-brain dysfunction.

Cortisol levels at admission (448 ± 813 versus 211 ± 122 nmol/L, *P* = 0.013) and over four days (*P* = 0.007, Figure 1) were higher in patients who developed brain dysfunction than non-brain dysfunction after adjusting with age and gender. Cortisol levels were higher in ICU nonsurvivors than survivors (510 ± 352 versus 248 ± 187 nmol/L, *P* = 0.05) but were not different between delirium and coma.

A cortisol cut-off value of 232 nmol/L determined brain dysfunction after sedation withdrawal with a sensitivity 59% and a specificity 63%. An age cut-off value of 57 years determined brain dysfunction with sensitivity 83% and specificity 63%.

**Figure 2:**
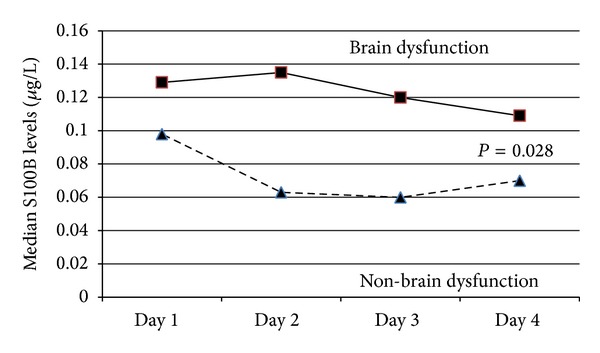
Patients with brain dysfunction released higher S100B levels than non-brain dysfunction.

S100B was elevated in brain dysfunction but not in non-brain dysfunction patients (*P* = 0.028, Figure 2). Comatose patients had higher S100B levels than delirium patients (median (interquartile): 0.14 (0.11, 0.48) versus 0.1 (0.05, 0.37) *μ*g/L, *P* = 0.007).

Cortisol correlated with S100B levels at ICU admission (*r* = 0.32, *P* < 0.01) and with the SOFA score at the first 4 days (*r* = 0.55, *P* < 0.01). Neither cortisol nor S100B was correlated with age, serum creatinine, APACHE III or SOFA scores, and C -reactive protein (CRP), (all *P* value >0.05).

Two risk factors were associated with the development of brain dysfunction in sepsis: cortisol levels at ICU admission (OR: 2.34, 95% CI (2.01, 3.22), *P* = 0.02) and the combination effect of cortisol with age (OR: 1.004, 95% CI (1.002, 1.93), *P* = 0.038). The AUC of the ROC curve of this global model to discriminate brain dysfunction was 89% (95% CI: 82, 95%) and remained unchanged when only delirium patients were included in the model.

## 4. Discussion

In our series of septic patients, brain dysfunction was present in one-half of the patients before sedation was started but increased to 84% of the patients after sedation withdrawal. Hence, sedation and/or other factors aggravated brain dysfunction at a certain time point in a number of patients during the sedation period. Benzodiazepines and opiates administration, although aggravating delirium in the critically ill patients, likely did not significantly influence the results as the number of patients who were sedated with midazolam and fentanyl and the length of sedation were not different in regard to brain dysfunction [16].

Our results suggested that cortisol was an associated-risk factor of brain dysfunction in sepsis. Pfister et al. previously showed that high cortisol levels were associated with S100B elevation and fatal brain dysfunction in a small number of patients with severe sepsis and septic shock [12]. Our results confirmed these observations in a larger cohort of septic patients, as cortisol levels were higher in patients with than patients without brain dysfunction. This could not result from a sole reaction to stress and illness severity because no association was found with the APACHE and SOFA scores or the degree of inflammatory response (as reflected by blood CRP concentrations). Also, cortisol levels decreased over time probably result from deficiency or exhaustion of the stimulating effect of adrenocorticotrophine hormone (ACTH) occurring in these patients [17].

It has been reported that cortisol could be harmful for the brain, especially at the hippocampus [18]. In rodents, cortisol impaired the neurons viability to insults (hypoxia, ischemia, or hypoglycemia) by inhibiting glucose uptake and utilization, exacerbating energy ATP depletion, and accumulated neurotoxic products over the course of few days [5, 6].

A cortisol cut-off value of 353–550 nmol/L was previously reported to be associated with delirium in stroke [8] and after cardiac surgery [9, 19]. However, we observed a lower cortisol cut-off value of 232 nmol/L associated with brain dysfunction in severe sepsis and this value was still remaining in our normal laboratory reference value (170–535 nmol/L). Hence, other factors should predispose the host brain to be more sensitive to the effect of cortisol. Sepsis with multiple organ dysfunction and aging could contribute to this process as these factors remained the main fatal ICU risk factors.

Sepsis aggravated ongoing brain dysfunction as a component of multiple organ dysfunctions which was reflected by S100B elevation and higher organ dysfunction SOFA scores, respectively. Different regions of the brain were affected in sepsis but the hippocampus was particularly sensitive to hypoxia and inflammation [20]. Moreover, the hippocampus regulates a negative feedback effect of cortisol release from the HPA [6, 21]. The dysfunction and damage of this part of the brain induced aberrant cortisol release, cognitive dysfunction, delirium and was implicated in the development of long term cognitive impairment in the sepsis survivors [17, 22–24]. In addition, the blood-brain barrier (BBB) breakdown in sepsis could worsen brain dysfunction by facilitating a crossing of cortisol, toxic amino acids and other proinflammatory mediators from the blood into the brain [25–27]. Using S100B as biomarker of brain injury BBB breakdown [28] we found that S100B elevation correlated with cortisol levels to support this hypothesis. Also, S100B was associated with brain dysfunction severity as higher levels were observed in fatal comatose than other patients.

Sepsis and brain dysfunction commonly targeted the aging patients [29, 30]. Patients who developed brain dysfunction were older and had higher cortisol levels than other patients. Elderly patients with preexisting brain disorder (dementia, atherosclerosis, and degenerative diseases) were more vulnerable to ischemia and to the harmful effect of proinflammatory cytokines or neurotoxic products than younger subjects [17, 31]. However, in our study, sepsis had more impact than preexisting brain disorders as it induced brain dysfunction in more than half of patients at ICU admission even before sedation was starting.

The logistic regression model also confirmed that cortisol levels associated with brain dysfunction were more pronouncing with age (older than 57 years old).

In addition to the above, the aging patients were chronically exposed to higher cortisol levels following an impairment of the negative feedback at the hippocampus with aging [32]. In these patients, cortisol could exacerbate neuronal dysfunction by energy deprivation, particularly at the hippocampus and the cortex, the parts of the brain where corticoid receptors are highly concentrated and sensitive to sepsis [6]. These observations raise concerns if the cortisol treatment should be selected in the aging patients with septic shock to prevent brain dysfunction, especially since it has been reported that a reduction of brain activity and cognitive dysfunction were observed after hydrocortisone administration in healthy volunteers [33].

In contrast with Pfister et al., we found that cortisol was more powerful than S100B to predict brain dysfunction and global ICU mortality. We previously showed that, due the short half-life time, S100B could only predict the early ICU nonsurvivors of sepsis [34]. Unlike S100B, cortisol was not solely a biomarker of brain dysfunction but was related with underlying life-threatening homeostasis disturbance of the host: abnormal HPA stress hormones response, glucose intolerance, immunodepression, and adrenal insufficiency [35–37].

S100B was sensitive to predict coma and severe brain injury (stroke, postanoxic encephalopathy, and head trauma) but more frequent measurements over a longer period than four days were probably necessary to diagnose delirium, which fluctuated over time [38–40]. Lacking of the statistical power with reduction of the predictive value of S100B to determine brain dysfunction could be another explanation as this biomarker was measured in half of patients.

Our study showed some limitations: we did not measure free serum cortisol which reflected its real biological activity although its advantage on total cortisol in sepsis was not confirmed by all authors [41]. Cortisol was not measured in the cerebral-spinal fluid although it was elevated in delirium patients [42]. Also, no direct harmful effect of cortisol on the brain or hippocampus could be demonstrated neither by neurophysiological studies nor brain imaging. We measured cortisol within a time interval but not at fixed time. However, as the circadian cortisol secretion dysfunction frequently occurred in sepsis this did not significantly influence our results [43].

S100B was not brain specific and could be released from sole stress or by other organs than the brain in shock [13, 44]. However, these factors likely did not significantly influence our results as no correlation was found with serum creatinine, APACHE, or SOFA scores. Moreover, it has been recently reported that the extracerebral source of S100B did not affect the levels of this biomarker in the serum after excluding major trauma or severe surgery [45].

## 5. Conclusion

Cortisol was an associated-risk factor of brain dysfunction in patients with severe sepsis and septic shock.

## Figures and Tables

**Table 1:** Characteristics at ICU admission and comorbidities.

Characteristics at ICU admission and comorbidities	*n* = 128
Age, years	65 ± 14
APACHE III score	83 ± 30
SOFA score	8 ± 4
CRP (mg/dL)	208 ± 129
Males/females, *n* (%)	83/45 (65/35)
Medical/surgical patients, *n* (%)	106/22 (83/17)
Bronchopneumonia, *n* (%)	93 (73)
Peritonitis, *n* (%)	22 (17)
Gram negative/positive, *n* (%)	65/42 (51/49)
Active smoking, *n* (%)	33 (26)
Obesity (BMI > 30), *n* (%)	28 (22)
Arterial hypertension, *n* (%)	53 (42)
Diabetes, *n* (%)	24 (19)
Alcohol abuse, *n* (%)	19 (15)
History of brain disorder, *n* (%)	29 (23)
History of Sepsis, *n* (%)	20 (16)

The continuous variables were represented as mean values ± standard deviation.

**Table 2:** Biomarkers levels, ICU clinical evolution, and outcome.

	All patients (*n* = 128)	Non-brain dysfunction (*n* = 21)	Brain dysfunction (*n* = 107)	*P*
Cortisol admission (nmol/L)	413 ± 757	211 ± 122	448 ± 813	0.013
Cortisol day 4 (nmol/L)	279 ± 256	314 ± 352	273 ± 238	0.441
S100B admission (*μ*g/L)	**0.13 (0.07, 0.28)**	0.97 (0.51, 0.18)	0.13 (0.06, 0.49)	0.188
S100B day 4 (*μ*g/L)	**0.13 (0.06, 0.2)**	0.08 (0.04, 0.13)	0.12 (0.08, 0.24)	0.038
Mechanical ventilation, days	16 ± 14	12 ± 11	15 ± 14	0.109
Inotropics length, days	6 ± 5	4 ± 2	6 ± 5	0.191
SOFA score for the first 4 days	8 ± 4	6 ± 4	9 ± 4	0.006
Sedation, days	9 ± 7	8 ± 6	10 ± 8	0.315
Midazolam, *n* (%)	80 (62)	12 (57)	68 (64)	0.579
Fentanyl, *n* (%)	75 (59)	13 (62)	62 (58)	0.736
Propofol, *n* (%)	48 (37)	9 (43)	41 (38)	0.696
Nosocomial infection, *n* (%)	67 (52)	5 (24)	62 (58)	<0.01
Shock recurrence, *n* (%)	37 (29)	1 (5)	36 (34)	<0.01
CINM, *n* (%)	64 (50)	4 (19)	60 (56)	0.003
ICU mortality, *n* (%)	69 (54)	3 (14)	66 (62)	<0.01

Continuous variables were represented as mean values ± standard deviation.

S100B levels were represented as median (interquartile) due to skewed values.

CNIM: critical illness neuromyopathy.
